# Synoviocyte Derived-Extracellular Matrix Enhances Human Articular Chondrocyte Proliferation and Maintains Re-Differentiation Capacity at Both Low and Atmospheric Oxygen Tensions

**DOI:** 10.1371/journal.pone.0129961

**Published:** 2015-06-15

**Authors:** Thomas J. Kean, James E. Dennis

**Affiliations:** 1 Department of Orthopaedics, Case Western Reserve University, Cleveland, OH, United States of America; 2 Matrix Biology Program, Benaroya Research Institute at Virginia Mason, Seattle, WA, United States of America; 3 Orthopedic Surgery, Baylor College of Medicine, Houston, TX, United States of America; University of California, San Diego, UNITED STATES

## Abstract

**Background:**

Current tissue engineering methods are insufficient for total joint resurfacing, and chondrocytes undergo de-differentiation when expanded on tissue culture plastic. De-differentiated chondrocytes show poor re-differentiation in culture, giving reduced glycosaminoglycan (GAG) and collagen matrix accumulation. To address this, porcine synoviocyte-derived extracellular matrix and low (5%) oxygen tension were assessed for their ability to enhance human articular chondrocyte expansion and maintain re-differentiation potential.

**Methods:**

Porcine synoviocyte matrices were devitalized using 3 non-detergent methods. These devitalized synoviocyte matrices were compared against tissue culture plastic for their ability to support human chondrocyte expansion. Expansion was further compared at both low (5%), and atmospheric (20%) oxygen tension on all surfaces. Expanded cells then underwent chondrogenic re-differentiation in aggregate culture at both low and atmospheric oxygen tension. Aggregates were assessed for their GAG and collagen content both biochemically and histologically.

**Results:**

Human chondrocytes expanded twice as fast on devitalized synoviocyte matrix vs. tissue culture plastic, and cells retained their re-differentiation capacity for twice the number of population doublings. There was no significant difference in growth rate between low and atmospheric oxygen tension. There was significantly less collagen type I, collagen type II, aggrecan and more MMP13 expression in cells expanded on synoviocyte matrix vs. tissue culture plastic. There were also significant effects due to oxygen tension on gene expression, wherein there was greater collagen type I, collagen type II, SOX9 and less MMP13 expression on tissue culture plastic compared to synoviocyte matrix. There was a significant increase in GAG, but not collagen, accumulation in chondrocyte aggregates re-differentiated at low oxygen tension over that achieved in atmospheric oxygen conditions.

**Conclusions:**

Synoviocyte-derived matrix supports enhanced expansion of human chondrocytes such that the chondrocytes are maintained in a state from which they can re-differentiate into a cartilage phenotype after significantly more population doublings. Also, low oxygen tension supports GAG, but not collagen, accumulation. These findings are a step towards the production of a more functional, tissue engineered cartilage.

## Introduction

Human articular cartilage has long been known to have a poor repair capacity [1]. Cartilage consists of a largely avascular, hypocellular, collagen and glycosaminoglycan (GAG) matrix [2]. Current orthopedic clinical practice attempts to relieve pain, aid repair or delay damage using non-steroidal anti-inflammatories, steroid injections, or hyaluronic acid injections. For large lesions, or those that do not respond to these treatments, surgeons have several options, including: microfracture, mosaicplasty [3], autologous chondrocyte implantation (ACI) [4], matrix assisted autologous chondrocyte implantation (MACI) [5], or, finally, total joint replacement. There are advantages and disadvantages to each technique. Microfracture and mosaicplasty have the advantage of being performed in a single surgery, but are only applicable to small lesions, and microfracture often results in a fibrous repair [5]. Both ACI and MACI are autologous cell transplantation techniques, which have the disadvantage of requiring two surgical procedures; cartilage biopsies are taken from low load-bearing regions, grown in culture and then implanted. However, ACI is a generally accepted technique, with good long-term outcomes when assessed by patient scoring systems [6, 7]. In an objective assessment of chondrogenesis, biopsies of 406 ACI patients showed hyaline cartilage in only 14.9% of patients, mixed hyaline and fibrous cartilage in 27.5%, fibrocartilage in 47.7% and fibrous repair in the remaining 9.9% [8]; similar results have been reported elsewhere [9]. This lack of hyaline cartilage formation could be due to the limited ability of human chondrocytes to expand on tissue culture (TC) plastic and their progressive loss of chondrogenic phenotype [10–12] and decreased capacity to re-differentiate [13]. Without the formation of hyaline cartilage that is able to provide mechanical support during joint function, the long-term prognosis for these repaired joints is poor. In addition, joints with even larger lesions are unlikely to see any clinical benefit from these procedures. As such, it is currently unfeasible for a human total joint to be resurfaced with autologous chondrocytes without improved expansion conditions that preserve chondrogenic potential.

Synoviocyte matrix has previously been shown to provide an environment that enhanced the growth and reduced the dedifferentiation of porcine articular chondrocytes [14], but this has not been shown for human chondrocytes. Previous work on synoviocyte matrices has used detergent as a decellularization method [14, 15]. However, detergent decellularization removes some extracellular matrix and disrupts collagen [16], and it is also difficult to completely remove detergents, which can have detrimental effects on cell viability [17]. This report describes the assessment of three non-detergent methods of devitalization [16], including a novel ethanol-based devitalization, of synoviocyte matrix for the expansion of human chondrocytes.

Low O_2_ (5%) tension during expansion culture has been reported to trend towards enhanced growth rate in rabbit chondrocytes [18], but to have no significant effect on human articular chondrocyte growth [19]. When cells are re-differentiated in low O_2_, chondrogenesis has been elevated in rabbit [18], bovine [20], and human [19, 21] articular chondrocyte cultures. However, it has also been shown to have negative effects on chondrogenesis in bovine articular chondrogenesis [22] and highly donor dependent results with human bone marrow MSCs [23]. Due to the conflicting results found in the study of chondrocyte expansion under low or atmospheric oxygen tensions (Table 1) [24], we considered oxygen tension to be an important parameter to investigate. Therefore, low O_2_ conditions were tested in conjunction with chondrocyte expansion on synoviocyte matrices and their subsequent re-differentiation in aggregate culture.

**Table 1:** Effect of reduced oxygen tension on *in vitro* human chondrogenesis.

Cells	Differentiation Format	GAG		Collagen		Gene					Harvest time	Expansion	Differentiation O_2_ tension (%)	Ref
		*BC	**H	*BC	**H	Sox9	Col1	Col2	Col10	ACAN	(d = days, wk = weeks)	Passages (P) and O_2_ tension (%)		
ACs	Micropellets	+	=		+	-	=	+	-	+	d4, d7, d11, d14	P3 2%	2, 20%	[21]
ACs	Pellet	=	=	na	=	na	=	+	=	+	d7, d14, d28	P3 5, 20%	5, 20%	[19]
ACs	Pellet	+	+	+	+	na	na	+	na	+	2wk, 4wk	P2 5, 19%	5, 19%	[49]
ACs	Pellet	+	na	-	=	na	+	+	+	+	d14	P1-3, 20%	2, 20%	[48]
ACs	Alginate beads	=	na	=	na	na	na	na	na	na	d1, d2, d4	P3 5%	<1, 2, 5, 21%	[52]
ACs	Alginate beads	+	na	na	na	+	=	+	na	+	d7,d14,d28	P3 20%	5, 20%	[53]
ACs	Alginate beads	na	na	na	na	=	=	=	na	=	d5	P3 21%	5, 20%	[54]
ACs	Alginate beads	na	na	na	na	+	=	+	na	+	d21	P2-3 21%	5, 21%	[55]
ACs	fibrin glue, genipin cross-linked	=	=	-	=	=	=	+	na	=	d1, 2.5wk, 7wk	P0 21%	5, 21%	[56]
ACs	MPEG-PLGA	na	na	na	na	+	=	+	na	+	d1, d2, d6	P2 21%	1, 5, 21%	[57]
ACs	Monolayer	na	na	+	na	+	na	+	na	+	d3	P0-3 20%	1, 20%	[58]
AC explants	Explants	na	na	na	na	+	na	+	=	+	d3	P0-3 20%	1, 20%	[59]
ACs	Type I and III collagen sponge	+	na	+	na	+	-	+	=	+	d0, d3, d7, d14	P1 21%	3, 21%	[60]
BM MSCs	Alginate beads	na	+	na	=	+	+	+	+	+	d1, d3, d7, d21	P5 21%	5, 21%	[61]
BM MSCs	Pellet	+/-	+/-	+/-	+/-	na	+/-	+/-	+/-	na	4wk	P2-4 21%	5, 20%	[23]
NCs	Pellet	+	+	na	=	na	na	na	na	na	d21	P2 21%	1, 5.25, 21%	[62]

Assessment of the effect of reduced oxygen tension on chondrogenesis: several of the manuscripts were comparing the effects of factors other than oxygen tension. Where the normoxic/hypoxic responses were not compared, assessment was made comparing the data presented for normoxic and hypoxic conditions in the same format; + indicates overall positive results,—indicates overall negative results and = indicates overall no significant effect, the +/- indicates that different donors responded differently;

*BC = biochemical assay,

**H = histological assessment;

ACs = articular chondrocytes, BM MSCs = bone marrow mesenchymal stem cells, NCs = nasal chondrocytes; for gene expression, an increase in expression of Sox9, Collagen type 2 (Col2) and Aggrecan (ACAN), and a decrease in expression of Collagen type1 (Col1) and collagen type 10 (col10) were considered positive in terms of articular chondrogenesis.

The present study looked at two factors vital to improved cartilage tissue engineering: 1) increased cell expansion; and 2) maintenance of chondrogenic potential. It was hypothesized that devitalized synoviocyte matrix would both increase the expansion of human chondrocytes and preserve their chondrogenic potential. Initial studies determined that detergent-based devitalization methods, even at low concentrations or after extended washes, caused the majority of the synoviocyte cell matrix to detach, and did not improve human chondrocyte expansion. To address these problems, three alternative synoviocyte extraction methods were assessed: freeze/thaw; freeze/thaw plus sonication; dry ice-cooled ethanol. All three showed high retention of the devitalized synoviocyte matrix and supported human chondrocyte growth. These three methods were compared to identify the one which produced the greatest population expansion while maintaining differentiation potential. It was also hypothesized that low O_2_ tension would support chondrocyte growth and re-differentiation. It was further hypothesized that the combination of the synoviocyte matrix and low O_2_ tension would have an additive effect on cell growth and re-differentiation. Re-differentiation was assessed by GAG and collagen content biochemically, and by GAG and type II collagen staining histologically. Re-differentiation into hyaline- like articular cartilage is defined by GAG and collagen accumulation, specifically type II collagen.

In summary, it was hypothesized that synoviocyte matrix expansion would support human chondrocyte chondrogenesis over more population doublings (PDs); that low O_2_ tension would support chondrogenesis; and that the combination of the synoviocyte matrix expansion and low O_2_ tension would have a cumulative effect on chondrogenesis.

## Materials and Methods

### Ethics Statement

Human chondrocytes were obtained from discarded tissue from total joint replacement surgeries with written informed consent under an IRB approved protocol: IRB # IRB08-00104 of The Metro Health System, Cleveland, OH. Synovial tissue was obtained from humanely euthanized sows after their use in surgical training (unpublished, not connected with this research). The surgical training lab was conducted under an IACUC approved protocol. Pigs were used in a terminal procedure and euthanized by lethal injection of Euthasol (pentobarbital sodium and phenytoin sodium) per protocol at the end of the lab, but prior to tissue harvesting (Case Western Reserve University and University of Washington).

### Synoviocyte isolation, culture and devitalization methods

Synoviocytes were isolated and cultured much as previously described [15]. Briefly, synovial membrane tissue was isolated from the knees of ~6-month-old sows within 2 h postmortem using sterile surgical technique (n = 5). The isolated membrane was then minced and digested with bovine testicular hyaluronidase (Sigma; 660 Units/ml in DMEM-LG) for 15 min at 37°C, centrifuged (500 RCF, 5 min) and the supernatant discarded. The tissue was then digested with trypsin/EDTA (Gibco; 0.25% (w/v)) for 30 min at 37°C, centrifuged (500 RCF, 5 min) and supernatant discarded before a final digestion in type II collagenase (Worthington Biochemical Corp.; 583 Units/ml) for 3 h at 37°C. The resulting solution was passed through a 70 μm filter and centrifuged (690 RCF; 10 min). Cells were counted and flasks seeded at 3,000 cells/cm^2^ and cultured in DMEM-LG, 10% (v/v) FBS, Pen/Strep (100 Units/100 μg/ml) until confluent. At the end of primary culture (P0) and second passage (P2) stocks were made by controlled rate freezing in FBS containing 10% (v/v) DMSO and stored under liquid nitrogen. For the production of synoviocyte matrix-coated flasks, P2 cells were thawed from frozen stocks and seeded on TC flasks (Corning) at 6,000 cells/cm^2^. They were grown in DMEM-LG + FBS (10% v/v) until 80–90% confluence (~7 days), and then switched to DMEM-LG + FBS (10% v/v) containing 50 μM ascorbate-2-phosphate for an additional 7 days. Fully confluent flasks were rinsed with PBS, the PBS removed and quick frozen on dry ice-cooled ethanol (190 proof). Flasks were either stored at -80°C for later devitalization or immediately devitalized by one of three methods: 1) 3 cycles of thawing/freezing (F-SCM); 2) 3 cycles of thawing/freezing followed by 1 min of sonication (S-SCM); 3) addition of dry ice cooled ethanol (200 proof) onto the synoviocyte matrix surface (30 s) on dry ice-cooled ethanol (E-SCM). The ethanol extract was discarded and the flask dried in a laminar flow sterile environment for approximately 30 min. The flasks were rinsed twice with PBS and then used for the culture of human chondrocytes.

### Human chondrocyte expansion

**Fig 1:**
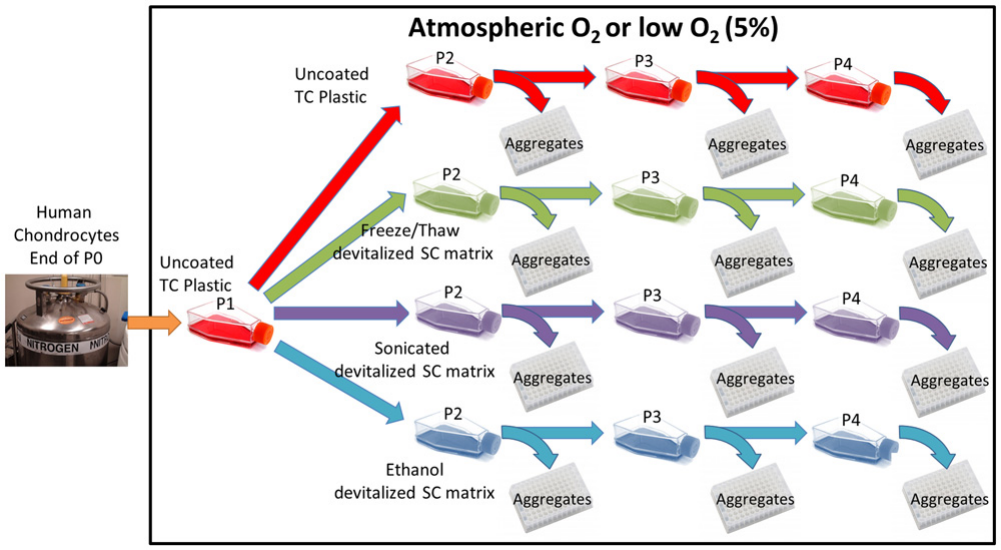
Experimental culture conditions overview. Human articular chondrocytes were thawed from frozen stocks and initially expanded on TC plastic before being split between each of the surfaces (TC plastic, F-SCM, S-SCM and E-SCM) and expanded for 3 passages with aggregates made at each passage. All steps were conducted at either (20%) atmospheric or low (5%) O_2_ tension.

Human chondrocytes were thawed from the end of P0 stocks, rinsed in expansion medium (DMEM-LG with 10% v/v FBS [human MSC selected lot [24]] containing penicillin/streptomycin [Invitrogen]), centrifuged (500 RCF, 5 min), resuspended in expansion medium, and a trypan blue-stained aliquot was counted in a hemacytometer. Cells were seeded into T175 TC plastic flasks (Corning) at 1 x 10^6^ cells/flask at both low (5%) and atmospheric (20%) O_2_. At confluence (end of P1), flasks were trypsinized (0.25% w/v trypsin/EDTA), the trypsin neutralized with FBS, centrifuged (500 RCF, 5 min), trypan blue stained, counted and split between synoviocyte matrix-coated or uncoated flasks under the same O_2_ tension as the prior expansion at 6,000 cells/cm^2^ (Fig 1). At confluence (end of P2, P3, P4), flasks were trypsinized, viable cells counted using trypan blue and passaged to a new flask under the same conditions and aggregate cultures made (Fig 1). PDs were calculated from the point the cells were split between each condition using the formula PD = (log_10_ (harvested cell count)—log_10_ (seeded cell count)) x 3.32 and sequential passages summed to give cumulative population doublings [25].

### qPCR chondrocyte gene expression analysis

qPCR experiments were conducted in accordance with the minimum information for publication of quantitative real-time pcr experiment (MIQE) guidelines[26]. Human chondrocytes (7 donors) were thawed from end of P0 stocks, and expanded as described above except on T75 flasks and only comparing TC plastic with E-SCM (Fig 1). Flasks were harvested at day 6 (P1), day 11 (P2), day 16 (P3) and day 21 (P4) during the logarithmic growth phase with 1 flask being extracted for RNA analysis with TRK Lysis buffer (E.Z.N.A. Tissue RNA Kit, Omega Bio-Tek) and the lysate frozen on dry ice and stored (-80°C, 1–6 weeks). The other flask was trypsinized, counted and seeded into a new flask. Total RNA was isolated from the lysates after thawing on ice using column purification (Direct-zol RNA mini-prep, Zymo Research), as per the manufacturer’s instructions with on-column DNA digest (DNase I, Zymo Research). RNA purity was analyzed by 260 nm / 280 nm ratio (Tecan Nanoquant) and degradation assessed using an Agilent Bioanalyzer (Genomic and RNA Profiling Core at Baylor College of Medicine). cDNA was synthesized from 500 ng of RNA using a Superscript III First Strand RT-PCR kit with oligo(dT)_20_ priming, according to the manufacturer’s instructions (Invitrogen). Quantitative real-time PCR was performed on a LightCycler Instrument (Roche) (2 μl of cDNA at a 1:8 dilution; with 16.5 μl of a mix containing 9.6 μl water (Roche), 2 μl of forward and reverse primers (50 nM), 2 μl Master mix (LightCycler FastStart DNA Master SYBR Green I, Roche), 2.4 μl MgCl_2_ (25 mM, Roche) with 0.4 μl of Uracil-DNA Glycosylase [Roche]). Cycling parameters were: 95°C for 10s then 45 cycles of 95°C 10s, 60°C 7s, 72°C 20s, followed by melt curve analysis. Seven reference genes (Ribosomal 18S, Glyceraldehyde 3-phosphate dehydrogenase, Beta-2 microglobulin, Hydroxymethylbilane synthase, Beta-actin, Tata-box binding protein and Hypoxanthine Phosphoribosyltransferase 1 (HPRT) were assessed for stability on a subset of samples. HPRT was determined to be the most stable across all conditions and therefore used as the internal control (reference gene) to normalize gene expression (primer sequences used are shown in S1 Table). All quantification cycles (Cq) were within the linear portion of log dilution analyses and >2 cycles higher than the negative control, melting temperatures were also consistent (S2 Table). Data for the comparison of expansion on TC plastic or E-SCM is presented as the expression of the target gene vs HPRT relative to the P1 sample on TC plastic. In the comparison of the effect of oxygen tension, each donor’s normalized gene expression at low oxygen tension was compared to its expression at atmospheric oxygen tension.

### Human chondrocyte re-differentiation—Aggregate cultures

Differentiation media contained DMEM-HG, ascorbate-2-phosphate (120 μM; Wako Pure Chemicals), dexamethasone (100 nM; Sigma), ITS + Premix (1% v/v; BD Biosciences), TGFβ1 (10 ng/ml; Peprotech), and sodium pyruvate (1 mM; Invitrogen) [27]. Trypsinized cells were resuspended in differentiation media and aggregate cultures made using 250,000 cells/aggregate (200 μl) seeded in each well of a polypropylene v-bottom 96-well plate. Plates were then centrifuged (600 RCF, 5 min), and placed in an incubator at the same O_2_ tension as that in which they were expanded. Media was changed on aggregates after 1 day, and every other day after that until harvest at day 21. At harvest, aggregates were weighed and either frozen (-80°C) for biochemical assays or fixed in neutral buffered formalin for histology.

### Biochemical analyses

Aggregates were thawed and papain digested (200 μl of papain digest buffer: 25 μg/ml papain (Sigma) in aqueous 2 mM cysteine (Sigma); 50 mM sodium phosphate (Sigma); 2 mM EDTA (Sigma); adjusted to pH 6.5 with 1 M HCl/NaOH) for 3 h at 65°C with vortexing every 30 min. Digests were then split between two tubes for either DNA/GAG assay [28] or hydroxyproline assay [29–32]. For the DNA/GAG assay, papain digestion was arrested by the addition of 200 μl of 0.1 M NaOH, incubated at RT for 30 min. This solution was then neutralized with 200 μl phosphate buffer (100 mM NaHPO_4_ (Sigma) pH 7.2; 0.1 M HCl). The DNA content was analyzed in triplicate against a standard curve produced with calf thymus DNA (Sigma) diluted in neutralized papain digest buffer using Hoechst dye (Hoechst 33258; Sigma; 0.667 μg/ml in 0.2 M phosphate buffer, pH 8). Fluorescence was read on a Fusion plate reader (Excitation 365 nm, Emission 460 nm; Packard Biosciences) using black 96-well plates (Greiner). GAG content was assessed from the same neutralized solution against a standard curve generated using chondroitin sulfate (Seikagaku) diluted in neutralized papain buffer. Colorimetric assays using Safranin-O were performed as previously described [28], and absorbance read at 536 nm (Optimax microplate reader, Molecular Devices). For the hydroxyproline assay, modifications to previously-described methods were made [29–32]. Briefly, 1 ml of HCl (6 M) was added to the digest and the sample hydrolyzed by incubation at 100–110°C overnight. Samples were then dried by incubation at 65–70°C for 1–2 days. Dried samples were then solubilized in 100 μl of 18 megaohm water (Nanopure, Thermo Scientific) and 20 μl transferred to a 96-well clear plate in triplicate. A standard curve of hydroxyproline diluted in papain buffer was also added. To these, 20 μl of 0.15 M CuSO_4_ and 20 μl of 2.5 M NaOH was added and incubated at 50°C for 5 min, followed by the addition of 20 μl of 6% (v/v) H_2_O_2_ and a further incubation at 50°C for 10 min. Plates were allowed to cool to RT before addition of 80 μl of 1.5 M H_2_SO_4_, then 40 μl of Ehrlich’s reagent (10% (w/v) *p*-dimethyl-amino-benzaldehyde (Sigma) in 60% (v/v) isopropanol, 18.2% (v/v) perchloric acid, 21.8% (v/v) megaohm water) and incubation at 70°C for 16 min. Following this, plates were allowed to cool to RT before analysis of absorbance (505 nm, Optimax microplate reader, Molecular Devices).

### Histological assessment

Aggregates, fixed in neutral buffered formalin (Azer Scientific), were paraffin-embedded and sectioned (5 μm). Sections were stained for GAG: 6 min in 0.1% (w/v) Safranin-O solution containing 1% (v/v) acetic acid and counterstained for 2 min in 1% (w/v) Fast Green containing 7% (v/v) acetic acid. For immunohistochemistry, unstained sections were de-paraffinized, outlined with a mini pap pen (Diagnostic Biosystems) and covered with pronase (1 mg/ml (Sigma) in PBS containing 5 mM CaCl_2_) and incubated at RT for 10 min. Sections were then rinsed with PBS, blocked with PBS/BSA 1% (w/v) (10 min RT), incubated with primary antibody overnight at 4°C (type II collagen: 1/500 in PBS/BSA 1% (w/v) [mouse monoclonal IgG1, raised against chicken collagen type II; II-II6B3 cell culture supernatant, DSHB]; type X collagen: 1/500 in PBS/BSA 1% (w/v) [mouse monoclonal, a kind gift from Dr. Gary Gibson, Henry Ford Hospital]; elastin: 1/400 in PBS/BSA 1% (w/v) [mouse monoclonal clone 10B8 raised against chick tropoelastin, MAB2505; Chemicon]). Following this they were rinsed with PBS and incubated for 1 h at RT with secondary antibody (Horse anti-mouse, 1/2000 in PBS/BSA 1% (w/v); BA-2000, Vector Labs), rinsed with PBS then incubated with streptavidin-conjugated horseradish peroxidase for 45 min at RT (1/400 in PBS/BSA 1% (w/v), SNN1004, Gibco), rinsed with PBS then incubated with peroxidase substrate (VIP substrate, Vectashield) for 10 min at RT. Slides were rinsed in distilled water and counterstained with Fast Green (0.0125% (w/v) in 24.25% (v/v) ethanol, 0.875% (v/v) acetic acid; Alfa Aesar) before rinsing in distilled water, and dehydration with ethanol then xylene, before drying and mounting (Permount, Fisher Scientific). Images were taken on a DME Upright brightfield microscope (Leica) at RT using a 10x 0.25 NA lens, using Leica Acquisition Software EZ (v1.7.0) with a Leica EC3 camera and background corrected and tiled using GNU Image Manipulation Program (GIMP 2.8.6).

### Statistical analyses

Comparisons between regression curves were made using a method equivalent to an Analysis of Covariance (ANCOVA, GraphPad Prism V.6.02). Comparisons of growth rates were made using one-way repeated measures analysis of variance (ANOVA) with Sidak’s correction for multiple comparisons (GraphPad Prism V.6.02). Matrix accumulation results were compared with repeated measures two-way ANOVA with Holm-Sidak’s correction for multiple comparisons (GraphPad Prism V.6.02). Relative gene expression values were compared between surfaces at each passage with a ratio paired t-test (GraphPad Prism V.6.02) using Bonferroni correction to indicate significance (i.e. *p* < 0.05/3). For comparison between oxygen tensions, ratio paired t-tests were used (GraphPad Prism V.6.02) using Bonferroni correction to indicate significance. Comparisons of gene expression with the control (P1 TC plastic expansion) were performed with Linear trend post-hoc analysis (GraphPad Prism V.6.0.2). Values quoted in the text are mean ± S.D.

## Results

### Expansion

**Fig 2:**
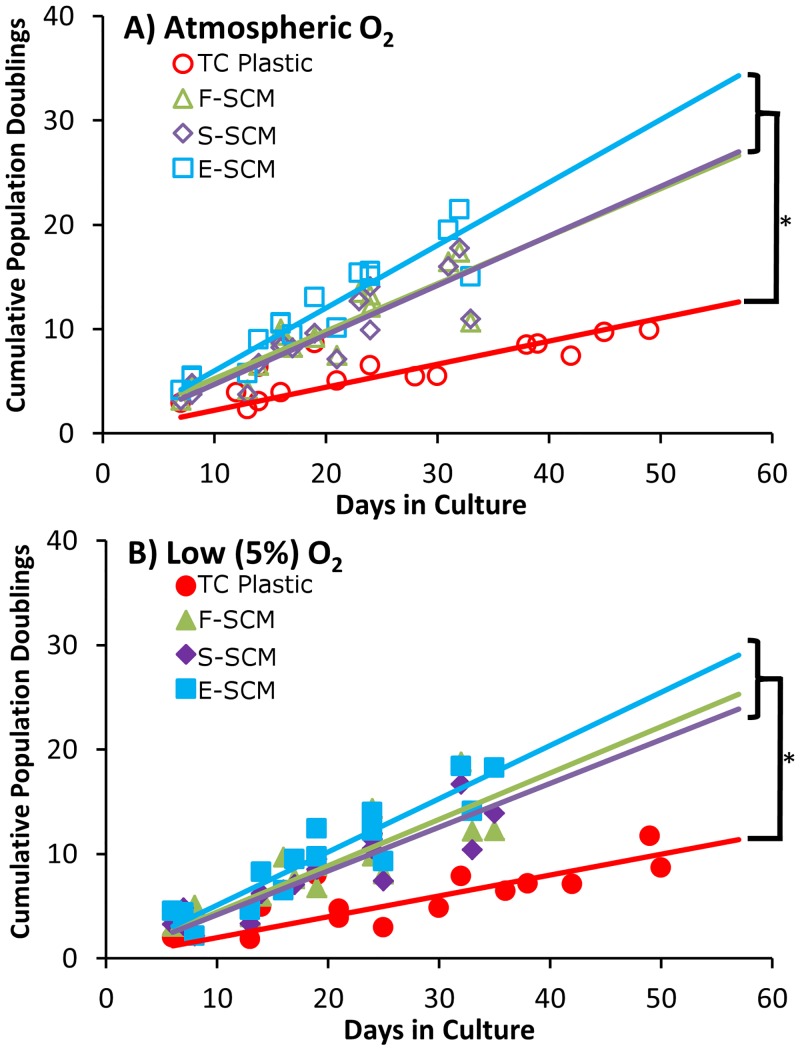
Human chondrocyte expansion on plastic and devitalized synoviocyte matrix. Cumulative population doublings; 5 independent experiments from 4 donors with 3 passages in each condition. A) treatment comparison at atmospheric O_2_. B) treatment comparison at low O_2_. Linear regressions from matrix expanded cells were significantly different compared to uncoated controls (*p* < 0.01).

**Fig 3:**
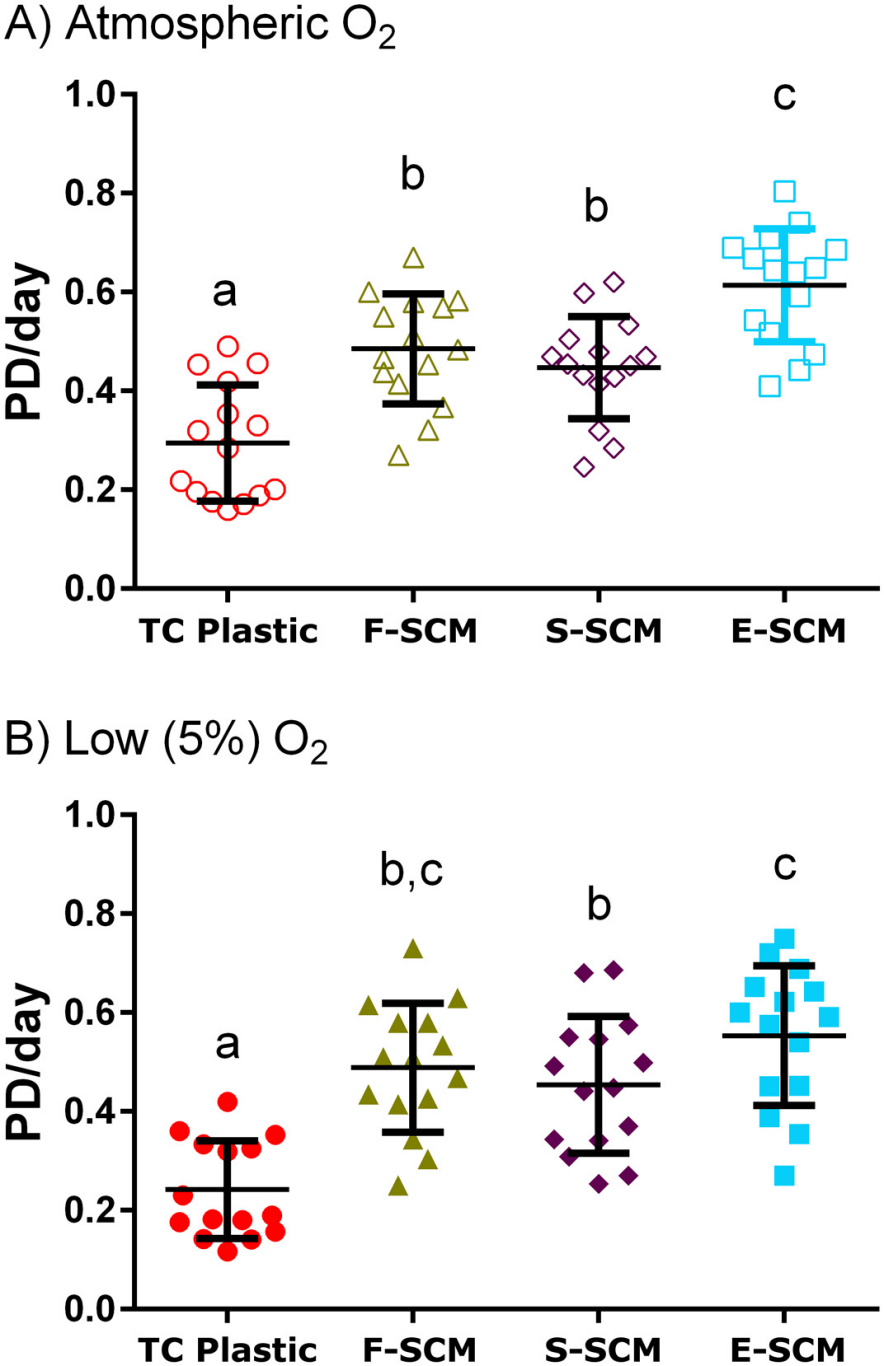
Population doubling rate on plastic and devitalized synoviocyte matrix. The population doubling rate from 5 independent experiments from 4 donors with 3 passages in each condition. A) treatment comparison at atmospheric O_2_. B) treatment comparison at low O_2_. Conditions which do not share the same letter were significantly different (*p* < 0.002).

We found significantly enhanced growth of human chondrocytes on all synoviocyte matrix-coated flasks compared to cells on TC plastic alone (uncoated) at both atmospheric (20%) O_2_ (Fig 2A) and low (5%) O_2_ (Fig 2B) conditions, regardless of the method used to devitalize the matrix. Although none of the growth curves on devitalized matrix were significantly different from each other, they were all significantly different from those on uncoated plastic (*p* < 0.01). The growth rate on matrix-coated flasks was approximately double that on uncoated flasks (Fig 3A and 3B) with all devitalized synoviocyte matrix-coated flasks yielding significantly greater cell numbers than uncoated flasks (*p* < 0.002). Devitalization using the dry ice-cooled ethanol method (E-SCM) resulted in the most consistent surface, with no detachment of the cell layer, giving a significant increase in growth rate at atmospheric O_2_ over that found with the other methods (F-SCM & S-SCM; Fig 3A). At low O_2_, this increase in growth rate over the two other methods was less pronounced and only statistically significant between the E-SCM and S-SCM groups (Fig 3B). Within each expansion surface, oxygen tension did not have a significant effect on growth rate (Figures A and B in S1 Fig). Control comparisons between human chondrocyte growth on TC plastic, and expansion on TC plastic coated with gelatin, collagen type I, or fibronectin showed no apparent morphological or proliferative benefit from the coatings (S2 Fig).

### Gene expression

**Fig 4:**
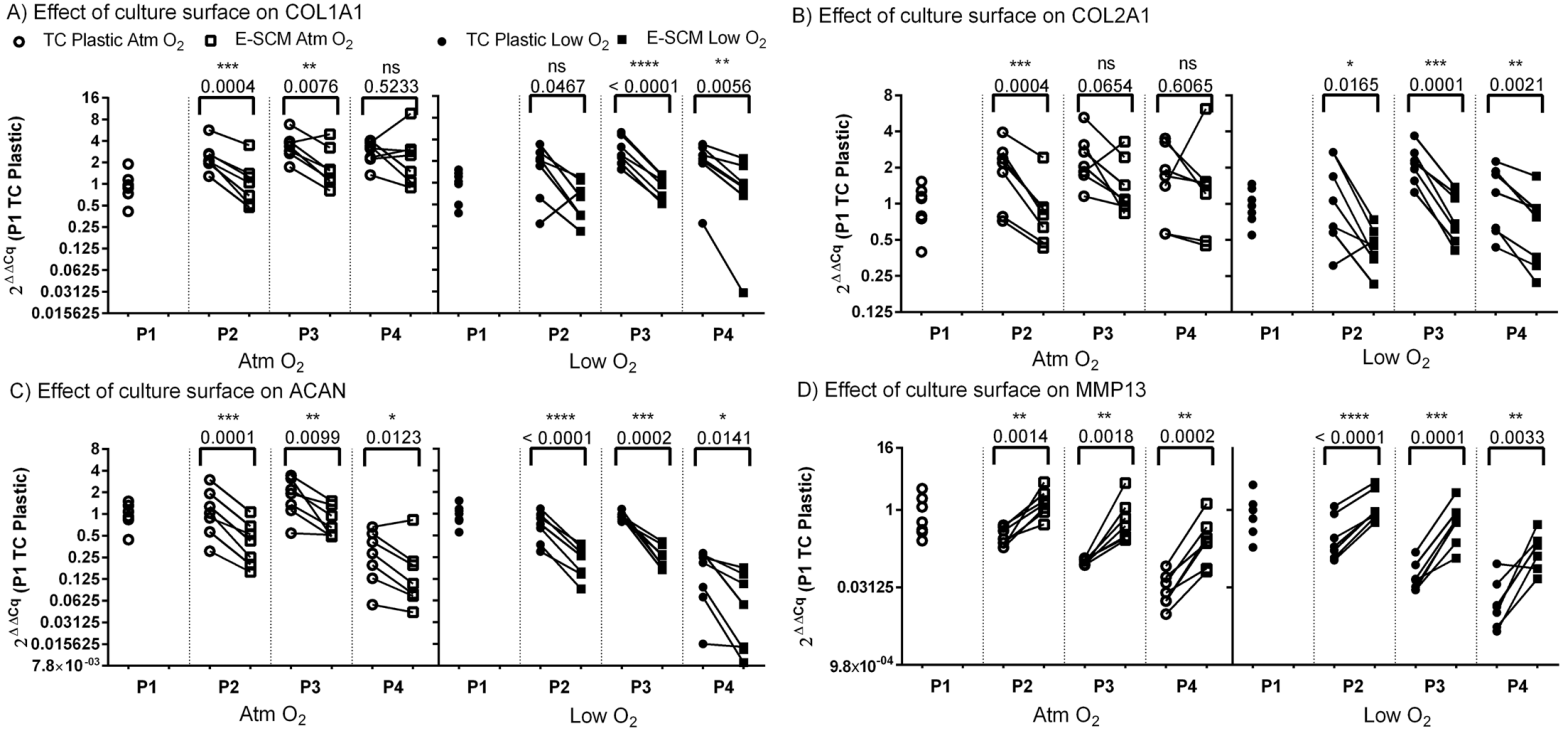
Effect of expansion culture surface on gene expression. Human chondrocytes from 7 donors were expanded on TC plastic or E-SCM at either atmospheric or low O_2_. Each point represents a donor, and lines connect the condition. A) Fold change of COL1A1 relative to P1 TC plastic expansion. B) Fold change of COL2A1 relative to P1 TC plastic expansion. C) Fold change of ACAN relative to P1 TC plastic expansion. D) Fold change of ACAN relative to P1 TC plastic expansion. *p* values and significance are indicated above comparisons.

RNA isolated from monolayer culture had high 260/280 nm ratio (2.03 ± 0.02) and good yield (377 ± 147 ng/μl giving 18.8 ± 7.4 μg total). RNA integrity numbers (RIN) also showed intact RNA (9.04 ± 0.83). Reference gene analysis showed HPRT to be the most stable across the conditions (S1 Table) and was similar for passage 1–3 with increased expression at P4 (Figure B in S3 Fig), and a linear trend was evident within each condition against population doublings (Figure A in S3 Fig). Further analysis showed that, within each passage, there was no significant difference in HPRT expression between the groups tested (Figures C-F in S3 Fig). Relative gene expression was therefore tested within passages, and comparison between passages was only made up to P3. Expansion on E-SCM significantly decreased collagen type I expression at both atmospheric and low oxygen tensions (Fig 4A). Collagen type I expression increased with passage on TC plastic (*p =* 0.0003 at Atm O_2_, *p* = 0.0010 at Low O_2_) with no significant trend on E-SCM. Type II collagen expression was also significantly lower when cultured on matrix at both oxygen tensions (Fig 4B). Type II collagen also showed a trend toward increased expression with passage on TC plastic (*p* = 0.0116 at Atm O_2_, *p* = 0.0075 at Low O_2_) with no significant trend on E-SCM. Type X collagen was unaffected by culture on synoviocyte matrix at either oxygen tension with no trend across passages (Figure A in S4 Fig). Aggrecan expression was significantly lower in chondrocytes cultured on synoviocyte matrix at both oxygen tensions (Fig 4C) with a negative linear trend on E-SCM at Low O_2_ (*p* < 0.0001). A statistically significant effect on SOX9 expression was only evident at P4 with an increase in expression on E-SCM over TC plastic (Figure B in S4 Fig). There was no significant effect on matrillin-1 expression under either oxygen tension (Figure C in S4 Fig) but the samples did appear to separate into two distinct levels of response at both oxygen tensions. MMP13 was significantly increased by expansion on E-SCM at both oxygen tensions (Fig 4D) with a negative linear trend on TC plastic at both oxygen tensions (*p* = 0.0049 at Atm O_2_, *p* = 0.0239 at Low O_2_) and no trend on E-SCM.

**Fig 5:**
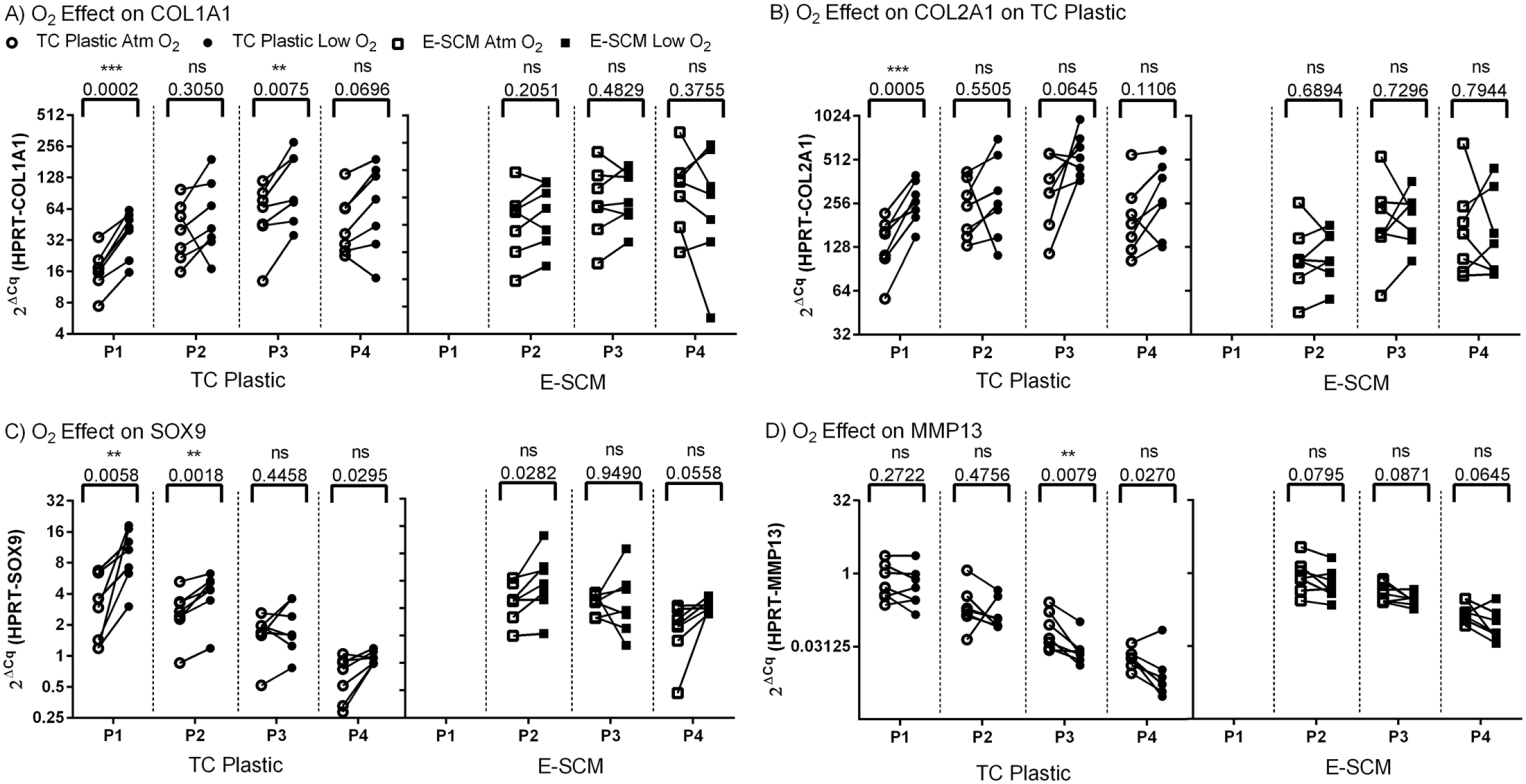
Effect of oxygen tension during expansion on gene expression. Human chondrocytes from 7 donors were expanded on TC plastic or E-SCM at either atmospheric or low O_2_. Each point represents a donor, and lines connect the condition. A) HPRT normalized COL1A1 expression. B) HPRT normalized COL2A1 expression. C) HPRT normalized SOX9 expression. D) HPRT normalized MMP13 expression. *p* values and significance are indicated above comparisons.

Examining effect of oxygen tension on gene expression, there was a significant increase in collagen type I expression on TC plastic at P1 and P3 but not on E-SCM (Fig 5A). A statistically significant increase in collagen type II expression was observed only at P1 on TC plastic, with later passages showing a trend towards increased expression on TC plastic but not on E-SCM (Fig 5B). Oxygen tension had no effect on collagen type X expression under any condition (Figure A in S5 Fig). At first passage on TC plastic, there was a significant increase in aggrecan expression at low O_2_ with trends evident at other passages, but not on E-SCM (Figure B in S5 Fig). SOX9 expression was significantly increased under low oxygen tension on TC plastic, but was not significantly different on E-SCM (Fig 5C). When plotted against population doublings (Figure A in S6 Fig), SOX9 showed a strong negative linear trend with population doubling on TC plastic (r^2^ = 0.23, *p* = 0.0001 at Atm O_2_; r^2^ = 0.52, *p* = 0.0002 at Low O_2_), while the negative slope was minimal on cultures on E-SCM. Oxygen tension had no significant effect on matrillin-1 expression (Figure C in S5 Fig). There was a significant decrease in MMP13 expression at lower oxygen tension at P3 on TC plastic, with trends apparent in other groups (Fig 5D). MMP13 expression showed a significant negative trend with increasing passage on TC plastic (Figure B in S6 Fig; r^2^ = 0.45, *p* = 0.0008 at Atm O_2_, r^2^ = 0.41, *p* = 0.0017 at Low O_2_).

### Re-differentiation

**Fig 6:**
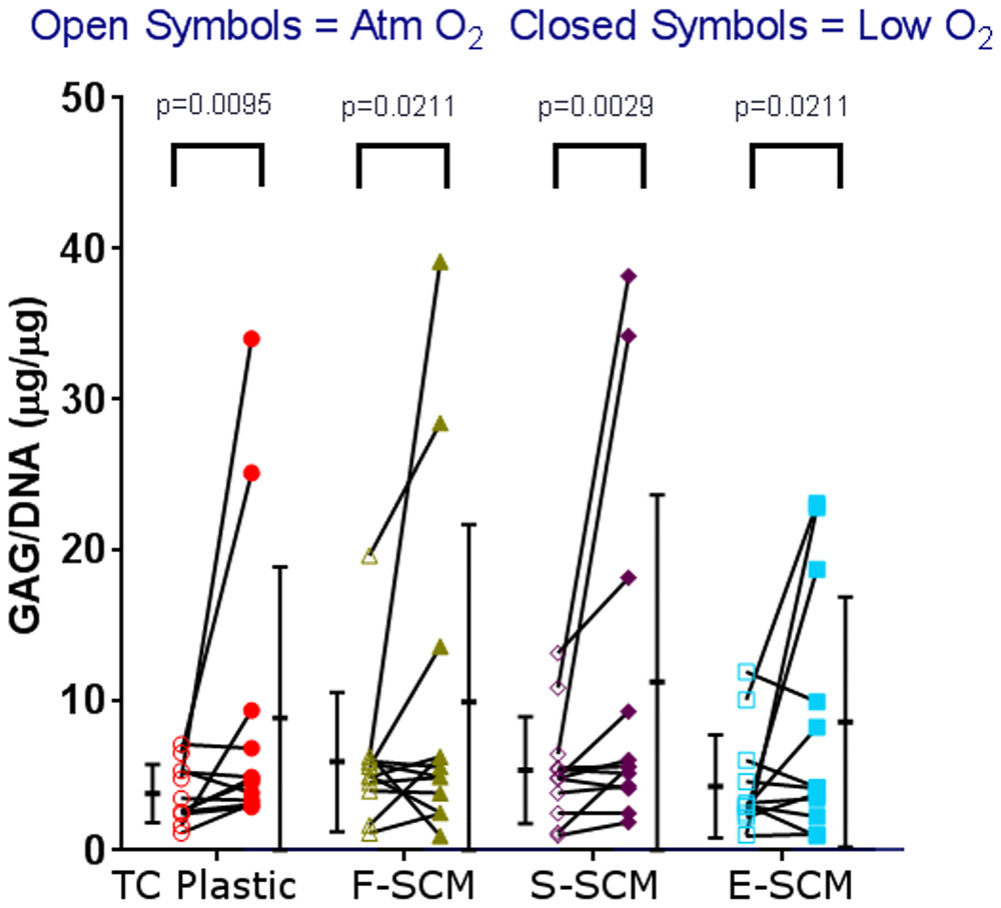
Glycosaminoglycan accumulation of differentially expanded human chondrocytes. Human chondrocyte aggregates from 4 donors were analyzed from 3 passages on TC plastic and SCM devitalized by 3 methods; aggregates were cultured at atmospheric (open symbols) and low (closed symbols) O_2_ tension. Each point represents the average of the aggregates assessed for that experiment (n = 1–3); beside these are the overall mean of all 3 passages for the 4 donors ± S.D., n = 12.

At atmospheric O_2_, cells that were expanded on devitalized matrix accumulated as much (F-SCM, E-SCM) or more (S-SCM) GAG than cells expanded on TC plastic alone, both in terms of total GAG and GAG/DNA (Fig 6). At low O_2_, there was no significant difference in GAG accumulation between the groups. A comparison between low and atmospheric O_2_ showed significantly more GAG accumulation at low O_2_ in all conditions (Fig 6). Regression analysis of the GAG/DNA content vs. PDs indicated that there was no significant difference in the slope of the lines for uncoated or coated flasks, but that there was a significant difference in the intercept, indicating that, for the same GAG content, cells expanded on devitalized matrix would have undergone a greater number of cell divisions while still producing the equivalent amount of GAG per DNA (Figures D and I in S7 Fig).

**Fig 7:**
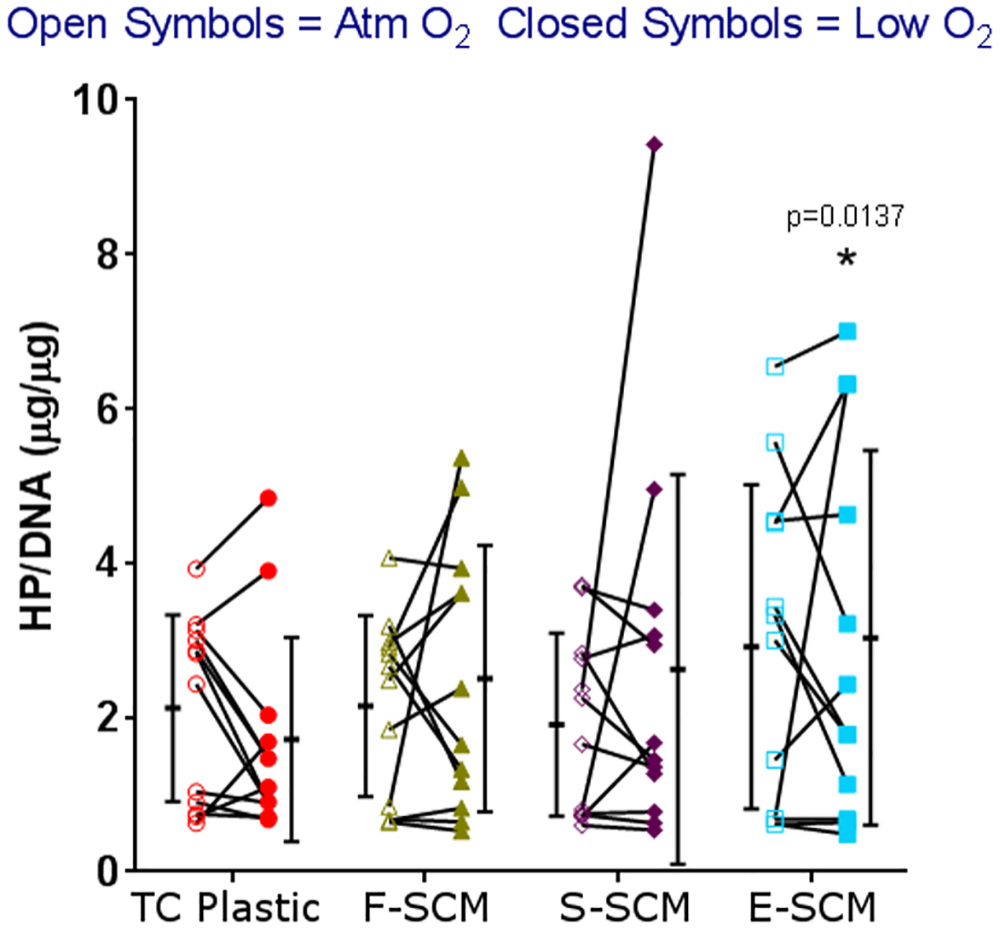
Hydroxyproline accumulation of differentially expanded human chondrocytes. Human chondrocyte aggregates from 4 donors were analyzed from 3 passages on uncoated flasks and synoviocyte matrix devitalized by 3 methods; aggregates were cultured at atmospheric (open symbols) and low (closed symbols) O_2_ tension. Each point represents the average of the aggregates assessed for that experiment (n = 1–3); mean and standard deviations are shown beside the individual data points, n = 12; * significantly different vs. uncoated at low O_2_ tension; there were no significant differences between hydroxyproline accumulation at different O_2_ tensions within the same culture conditions.

Cells which were expanded on E-SCM accumulated significantly more hydroxyproline (HP), corresponding to collagen, at both atmospheric and low O_2_ (Fig 7). Regression analysis of HP/DNA content vs. PDs indicated that coated flasks had similar slopes to uncoated flasks (or rates of loss of collagen content) but a higher intercept, indicating that at similar PDs, cells expanded on devitalized matrix would have greater collagen content (Figures E and J in S7 Fig).

**Fig 8:**
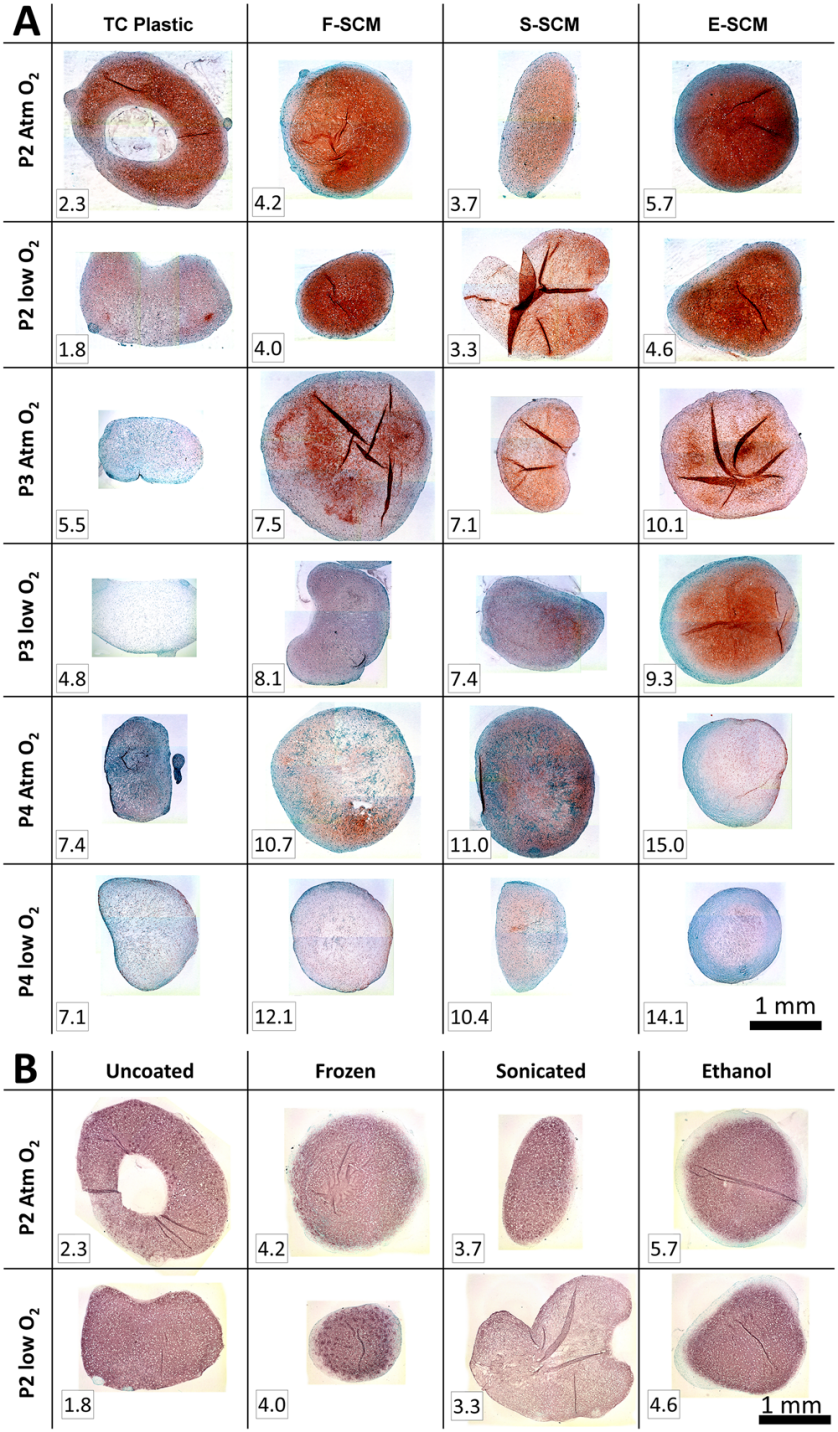
Histological assessment. A) Glycosaminoglycan accumulation in aggregates—Human chondrocyte aggregates from all three passages on the different substrates were fixed, embedded and sectioned then stained with Safranin-O/Fast Green for analysis. Representative images from one donor/set of experiments are shown. All images are at the same magnification (100x). Numbers in the bottom left corner for each image indicate the cumulated population doublings for those cells. B) Type II collagen immunohistochemistry—Human chondrocyte aggregates stained with anti-type II collagen antibody from the first passage (P1) are shown. Representative images from one donor/experiment are shown. All images are at the same magnification (100x). Numbers in the bottom left corner for each image indicate the cumulated population doublings for those cells.

Histologically, aggregates that were expanded on devitalized synoviocyte matrices showed Safranin-O staining that correlated qualitatively with the quantitative results seen by the GAG assay. Synoviocyte matrix-expanded cells in aggregates stained positively for GAG to a similar or greater extent despite having undergone significantly more PDs (Fig 8A). Immunohistochemistry for collagen type II showed similar or greater levels of staining in aggregates expanded on devitalized matrix, which was even more pronounced at low O_2_ (Fig 8B). Immunohistochemistry for secondary antibody alone, collagen type X and elastin were negative, and rabbit and human controls were positive (S8 Fig).

## Discussion

This study showed a significant improvement in human chondrocyte expansion with all 3 devitalized synoviocyte matrices compared to standard TC plates. In addition, low O_2_ promoted greater chondrogenesis during differentiation culture for both TC plastic- and synoviocyte matrix-expanded chondrocytes. The synoviocyte matrix-expanded chondrocytes, having undergone significantly more PDs, had levels of chondrogenesis similar to those for cells expanded on TC plastic.

This study also showed, in agreement with other studies, that expansion conditions can have a dramatic effect on chondrogenic capacity [18, 33–35]. Growth factor supplementation during expansion (basic fibroblast growth factor [bFGF]), epidermal growth factor, platelet derived growth factor, TGFβ1) has been investigated for its effect on human chondrocyte expansion [33, 35]. Both studies showed greater re-differentiation after bFGF-supplemented expansion, but only one achieved an increased growth rate [33]. It is likely that the devitalized matrix, in addition to providing a 3D surface that is amenable to chondrocyte growth, contains a mix of growth factors which will be investigated in future studies. It is interesting to note that co-culture experiments of synoviocytes and chondrocytes have shown a decrease in chondrogenesis [36, 37]. This was true for both bovine cartilage/synovium [37] and osteoarthritic human cartilage/synovium [36], so whatever detrimental effects living synoviocytes had on the chondrocytes was not present in the extracted matrix.

Some decellularization methods include a DNase treatment in the processing of the tissue [38]; this was not included in our protocol as DNase treatment after devitalization had no beneficial effect on the proliferation rate of the cells. Also, DNases are naturally present in FBS used in the expansion media. In our study, the synoviocyte-matrix is not acting as a conduit for transplantation as is the case with many decellularized tissues, so residual nuclear material is a diminished concern. To ensure synoviocytes were devitalized, serum-containing media was incubated on the devitalized matrix, and no growth or nuclei were discernible using Hoechst (3 μg/ml in PBS with Mg and Ca, 33342; Life Technologies) staining after 6 days (S7 Fig).

We concur with Pei and He [14] in their description of the synoviocyte matrix as “a tissue-specific three-dimensional microenvironment for *ex vivo* expansion of articular chondrocytes”. Similar expansion and differentiation results were achieved with human chondrocytes in our study as those previously described with porcine chondrocytes [14]. While Pei *et al*. have found detergent-based decellularization methods acceptable for porcine chondrocytes [14], porcine synovial stem cells [15], and human mesenchymal stem cells (MSCs) [39], we did not find that to be the case for human chondrocytes. This may have been due to residual detergent [17], the extensive loss of porcine synoviocyte matrix components, or from inherent differences between osteoarthritic human chondrocytes and healthy porcine chondrocytes. While the chondrocytes in this study were harvested from macroscopically normal areas, all donors were undergoing total joint replacement, so these cells represent a realistic sampling of the kinds of cells that would be used for ACI. Of the devitalization methods tested, dry ice cooled Ethanol-based devitalization proved to be the most consistent and facile method as it was rapid and showed little loss of the cell matrix layer. TC plastic expansion is known to de-differentiate chondrocytes, their ability to redifferentiate diminishing with increased passage number. In this study, we observed that the cells on synoviocyte matrix-coated plastic still lost the ability to re-differentiate, but the curve has been shifted to the right to give greater PDs (5–10 depending on the metric), with the same amount of GAG and collagen. For example, in the TC plastic vs. E-SCM samples at atmospheric O_2_, at 5 μg GAG/DNA the TC plastic expanded sample has undergone 3.3 PDs while the E-SCM expanded sample has gone through 10.5 PDs. From an average Carticel biopsy of 180,000 to 455,000 cells [4], TC plastic flasks could yield 1.8–4.4 x 10^6^ cells with chondrogenic potential, whereas E-SCM flasks could yield 2.6–6.4 x 10^8^ cells with equivalent chondrogenic properties.

The effect of the matrix support on gene transcription has given some interesting insights into chondrocyte biology. While many mature chondrocyte genes were downregulated during expansion on synoviocyte matrix, they clearly rebound during differentiation. This highlights an important point: Chondrocyte expansion is a separate function from differentiation, and seeking to induce the expression of mature chondrocyte genes during expansion might be a misstep. These studies clearly showed that the culture surface has a dramatic effect on gene expression, and a relationship between the surface and oxygen tension was also evident. The diminished changes with passage observed between the control P1 condition and increased passages on E-SCM support the hypothesis that the chondrocytes cultured on E-SCM are maintained in a state from which they can redifferentiate more readily.

The effect of oxygen tension on gene expression during expansion was more pronounced at earlier passages and often abrogated by E-SCM expansion. This dulled effect is unlikely to be due to the increased proliferation rate causing hypoxia. Although the cells would have increased oxygen consumption, the diffusion rate of oxygen is high *in vitro* [40]. An increase in collagen type II expression was observed with increasing passage along with a concurrent decrease in SOX9 expression; this was most evident on TC plastic, which presents an interesting dichotomy. Aigner *et al*. [41] found that SOX9 did not correlate with COL2A1 expression in adult human articular chondrocytes, which was proposed to be due to post-translational events/subcellular localization and could also be valid reasoning in our studies. In contrast to the study by Lin *et al*. [42], we found increased levels of COL2A1 and COL1A1 and no effect on ACAN with increasing passage. This disparity could be due to the use of confluent cultures or the use of GAPDH as a reference gene. Ma *et al*. also report decreased COL2A1 expression with increased passage, but no data is reported for ACAN or COL1A1 [11]. This difference could also be due to expansion conditions as Ma *et al*. cultured cells at a high density (25,000 cells/cm^2^) and did not report population doublings. Our choice of timing was made with the effect of culture confluence in mind, such that cells were harvested before confluence was achieved on either TC plastic or E-SCM, ensuring the cells were in as similar a proliferative stage as possible. It has been suggested that the relative expression of type II collagen/type I collagen is critical in terms of articular chondrogenesis [43]. Surprisingly, in our study, a relatively high expression of collagen type II was maintained throughout the expansion on both TC plastic and E-SCM and was even seen to increase compared to P1 on TC plastic. A similarly high expression of type II collagen was observed when cells were cultured on cartilage matrix [44]. A comparison of the normalized data demonstrates that a high COL2A1/COL1A1 ratio was maintained throughout expansion culture (Fig 5A and 5B).

Although oxygen tension has long been realized to be important in chondrogenesis [45], its mechanism and chondrogenic outcomes are not clear [46] (Table 1). In contrast to Egli *et al*.[47], Qu *et al*. [22] and Schrobback *et al*.[19], but in agreement with Markway *et al*.[48] and Strobel *et al*.[49], re-differentiation of chondrocytes under low O_2_ increased GAG accumulation within the aggregates. No significant increase in collagen content was found in cells cultured and re-differentiated at low O_2_, which is in contrast to the results of Strobel *et al*.[49] but in agreement with Markway *et al*.[48]. However, an increase in collagen deposition in aggregates from cells that had been cultured on E-SCM was found. This, in addition to the collagen type II staining, could point towards an enhancement of chondrogenic capacity which was lost through culture on TC plastic. It is clear that the cell type, species, expansion conditions, time in culture and differentiation conditions are all important. Concentrating on only human cells, the overall impression is that low oxygen tension is beneficial in one or more outcome measures, especially for articular chondrogenesis (Table 1). However, some groups have seen negative effects of low oxygen tension, with others observing no significant effects, on one or more outcome measures. It is clear that more work is needed to investigate this important parameter in tissue culture.

In contrast to MSC chondrogenesis, where type X collagen and hypertrophy are commonly reported [27, 50], we observed no type X collagen protein expression in any of our chondrocyte cultures and very low relative gene expression. This is supportive of the use of chondrocytes as the cell of choice for tissue engineering of articular cartilage.

Donor and passage number were significant sources of variability within this study in terms of both gene expression and matrix synthesis. Large donor variability has been described by others [23, 51], with over a 1,000-fold difference in collagen gene expression at the end of first passage, which was not related to age or gender. Despite this, synoviocyte matrix coated flasks gave significantly more cells with all donors. Ideally, we would also have been able to compare between aggregates made from cells expanded on the different surfaces at the same number of PDs. A close approximation to this can be seen in the Safranin-O histology (Fig 6A) where, at P1, E-SCM-expanded cells at atmospheric O_2_ have undergone 5.7 PDs and, at P2, TC plastic-expanded cells have undergone 5.5 PDs, and there is significantly greater Safranin-O staining for the E-SCM-expanded cells. The lack of GAG staining in the later passage supports the work presented by Giovanni *et al*., in which a 3.6–4.2 PD limit for chondrogenic capacity of human chondrocytes is suggested [13]. However, expansion on synoviocyte matrix seems to extend this limit to upwards of 8 PDs.

These data clearly indicate that, relative to TC plastic, synoviocyte-derived matrix supports enhanced expansion of human-derived chondrocytes, such that the chondrocytes are maintained in a state from which they are able to re-differentiate into a cartilage phenotype after significantly more PDs.

## Supporting Information

S1 Table Assessment of reference genes for qPCR. (DOCX)

S2 Table qPCR primer characteristics. (DOCX)

S1 Fig Effect of oxygen tension on human articular chondrocyte growth. A) Comparison of population doubling rate between each surface at atmospheric (open symbols) and low (5%) oxygen tension (closed symbols). B) qPCR expansion data, recapitulating the increased expansion rate and lack of effect of oxygen on human chondrocyte expansion.
(TIF)

S2 Fig Common tissue culture coated surface comparison. Analysis of growth and morphology on common surface coatings. Chondrocytes were seeded on each of the surfaces and tracked for 7 days. All images are at the same magnification (100x).
(TIF)

S3 Fig Reference gene analysis. HPRT was assessed across all conditions for its stability. A) Regression curve analysis vs. population doublings in each condition. B) Quantification cycle (Cq) assessment by passage, P4 was significantly different to all other passages (*p* < 0.0001, ANOVA). C) Comparison of Cq at P1 in each condition. D) Comparison of Cq at P2 in each condition. E) Comparison of Cq at P3 in each condition. F) Comparison of Cq at P4 in each condition. No significant differences were found for C-F.
(TIF)

S4 Fig Effect of expansion surface on the expression of COL10A1, SOX9 and MATN1. Human chondrocytes from 7 donors were expanded on TC plastic or E-SCM at either atmospheric or low O_2_. Each point represents a donor and lines connect the condition. A) Fold change of COL10A1 relative to P1 TC plastic expansion. B) Fold change of SOX9 relative to P1 TC plastic expansion. C) Fold change of MATN1 relative to P1 TC plastic expansion. *p* values and significance are indicated above comparisons.
(TIF)

S5 Fig Effect of oxygen tension during expansion on COL10A1, ACAN and MATN1. Human chondrocytes from 7 donors were expanded on TC plastic or E-SCM at either atmospheric or low O_2_. Each point represents a donor and lines connect the condition. A) HPRT normalized COL10A1 expression. B) HPRT normalized ACAN expression. C) HPRT normalized MATN1 expression. *p* values and significance are indicated above comparisons.
(TIF)

S6 Fig Correlation of SOX9 and MMP13 with cumulative population doublings. A) Correlations of SOX9 expression with cumulative population doublings (P1–P3) in each condition were made. B) Correlations of MMP13 expression with cumulative population doublings (P1–P3) in each condition were made.
(TIF)

S7 Fig Regression analysis for synoviocyte-matrix expanded chondrocytes. Regression analyses of biochemical measures against population doublings. Regressions were made combining all data from all 3 donors (n ≥ 23). A-E Atmospheric oxygen tension, F-J Low (5%) oxygen tension; A, F) Wet weight vs. population doublings; B,G) Total GAG (per aggregate) vs. population doublings; C,H) Total HP (per aggregate) vs. population doublings; D, I) Normalized GAG (GAG/DNA) vs. population doublings; E,J) Normalized HP (HP/DNA) vs. population doublings.
(TIF)

S8 Fig Immunohistochemistry controls. Representative sections of control tissues: rabbit (Rb) articular and auricular cartilage and human (Hu) articular cartilage. Representative sections from aggregates produced from cells expanded at atmospheric O_2_ on tissue culture plastic (uncoated) or E-SCM are also shown.
(PPTX)

S9 Fig Growth media catalysed DNA degradation. Synoviocyte-derived matrix was incubated with DMEM/FBS or PBS for 6 days; wells were then stained with Hoechst and imaged for nuclear staining (Fluorescence).
(TIF)
