# Correction: Synoviocyte Derived-Extracellular Matrix Enhances Human Articular Chondrocyte Proliferation and Maintains Re-Differentiation Capacity at Both Low and Atmospheric Oxygen Tensions

**DOI:** 10.1371/journal.pone.0138409

**Published:** 2015-09-14

**Authors:** Thomas J. Kean, James E. Dennis

S7 Fig is displayed incorrectly. It can be viewed below.

## Supporting Information

S7 Fig Regression analysis for synoviocyte-matrix expanded chondrocytes. Regression analyses of biochemical measures against population doublings. Regressions were made combining all data from all 3 donors (n ≥ 23). A-E Atmospheric oxygen tension, F-J Low (5%) oxygen tension; A, F) Wet weight vs. population doublings; B,G) Total GAG (per aggregate) vs. population doublings; C,H) Total HP (per aggregate) vs. population doublings; D, I) Normalized GAG (GAG/DNA) vs. population doublings; E,J) Normalized HP (HP/DNA) vs. population doublings.
(TIF)
