# Supplementary material for: Synoviocyte Derived-Extracellular Matrix Enhances Human Articular Chondrocyte Proliferation and Maintains Re-Differentiation Capacity at Both Low and Atmospheric Oxygen Tensions
Source: PLoS One. 2015 Jun 15;10(6):e0129961. doi: 10.1371/journal.pone.0129961 (PMC4468209; doi:10.1371/journal.pone.0129961)
Supplement: S1 Table — (DOCX) [file pone.0129961.s001.docx]

**S1 Table: Assessment of reference genes for qPCR**

| **Gene** | **Forward Primer** | **Reverse Primer** | **Accession number** | **RSD of subset (%)** |
| --- | --- | --- | --- | --- |
| RNA18S5* | CTCAACACGGGAAACCTCAC | TTATCGGAATTAACCAGACAAATCG | [NR_003286.2](http://www.ncbi.nlm.nih.gov/entrez/viewer.fcgi?db=nucleotide&id=225637497) | 17.84 |
| GAPDH | TCCTCTGACTTCAACAGCGACAC | ATACCAGGAAATGAGCTTGACAAA | [NM_002046.5](http://www.ncbi.nlm.nih.gov/entrez/viewer.fcgi?db=nucleotide&id=576583510) | 3.82 |
| B2M | AGCGTACTCCAAAGATTCAGGTT | TACATGTCTCGATCCCACTTAACTAT | [NM_004048.2](http://www.ncbi.nlm.nih.gov/nuccore/NM_004048.2) | 3.80 |
| HMBS | TGCAACGGCGGAAGAAAACA | AGATGGCTCCGATGG | [NM_000190.3](http://www.ncbi.nlm.nih.gov/nuccore/NM_000190.3) | 3.80 |
| ACTB | GCCAACCGCGAGAAGATGACC | CTCCTTAATGTCACGCACGATTTC | [NM_001101.3](http://www.ncbi.nlm.nih.gov/nuccore/NM_001101.3) | 6.12 |
| TBP | CTAAAGACCATTGCACTTCGT | GTTCGTGGCTCTCTTATCCTC | [NM_003194.4](http://www.ncbi.nlm.nih.gov/entrez/viewer.fcgi?db=nucleotide&id=285026518) | 4.69 |
| HPRT1 | ATTGACACTGGCAAAACAATGC | TCCAACACTTCGTGGGGTCC | [NM_000194.2](http://www.ncbi.nlm.nih.gov/nuccore/NM_000194.2) | 3.18 |
| *Andreea SI, Marieta C, Anca D (2008) AGEs and glucose levels modulate type I and III procollagen mRNA synthesis in dermal fibroblasts cells culture. Exp Diabetes Res 2008: 473603. | | | | |
