# Supplementary material for: Synoviocyte Derived-Extracellular Matrix Enhances Human Articular Chondrocyte Proliferation and Maintains Re-Differentiation Capacity at Both Low and Atmospheric Oxygen Tensions
Source: PLoS One. 2015 Jun 15;10(6):e0129961. doi: 10.1371/journal.pone.0129961 (PMC4468209; doi:10.1371/journal.pone.0129961)
Supplement: S2 Table — (DOCX) [file pone.0129961.s002.docx]

**S2 Table: qPCR primer characteristics**

| **Gene** | **Forward Primer** | **Reverse Primer** | **Accession number** | **Efficiency (%)** | **Highest Cq** | **Lowest Cq** | **Cq of -ve control** | **Amplicon length** | **Tm (°C)** |
| --- | --- | --- | --- | --- | --- | --- | --- | --- | --- |
| HPRT1 | ATTGACACTGGCAAAACAATGC | TCCAACACTTCGTGGGGTCC | [NM_000194.2](http://www.ncbi.nlm.nih.gov/nuccore/NM_000194.2) | 100 | 24.59 | 27.44 | >45 | 111 | 84 |
| COL1A1 | CCTGAGCCAGCAGATCGAGAACATCCG | GCAGGTCTCACCAGTCTCCATGTTGCAG | [NM_000088.3](http://www.ncbi.nlm.nih.gov/entrez/viewer.fcgi?db=nucleotide&id=110349771) | 100 | 17.46 | 24.11 | 36 | 187 | 86 |
| COL2A1 | TGGAGACTACTGGATTGACCCCAACCAA | TCTCGCCAGTCTCCATGTTGCAGA | [NM_001844.4](http://www.ncbi.nlm.nih.gov/entrez/viewer.fcgi?db=nucleotide&id=111118975) | 100 | 15.83 | 20.52 | 36 | 80 | 84 |
| COL10A1 | CATTCTCTAACTCTACCCCACCC | TCAAGAGAGGCTTCACATACGTT | [NM_000493.3](http://www.ncbi.nlm.nih.gov/entrez/viewer.fcgi?db=nucleotide&id=98985802) | 96 | 27.84 | 33.8 | >41 | 160 | 76 |
| ACAN | AGCCATCTCTACACGCTACACCCT | CCTTGTCTCCATAGCAGCCTTCCC | [NM_001135.3](http://www.ncbi.nlm.nih.gov/entrez/viewer.fcgi?db=nucleotide&id=256017258) | 97 | 19.92 | 26.88 | >40 | 200 | 92 |
| SOX9 | CGAACGCACATCAAGACGGAGCA | GGGGCTGTAGTGTGGGAGGTTGA | [NM_000346.3](http://www.ncbi.nlm.nih.gov/entrez/viewer.fcgi?db=nucleotide&id=182765453) | 107 | 22.205 | 26.97 | 35 | 111 | 89 |
| MATN1 | CCGCCGTAGCTTTACCATTTTAGTGA | CTCCCAAACGCCATTACACGCTCT | [NM_002379.3](http://www.ncbi.nlm.nih.gov/entrez/viewer.fcgi?db=nucleotide&id=206725426) | 110 | 18.76 | 34.88 | >41 | 99 | 87 |
| MMP13 | TGCCCTTCTTCACACAGACACTAACGAAA | GGCCACATCTACTATTCTTACCACTGCTC | [NM_002427.3](http://www.ncbi.nlm.nih.gov/entrez/viewer.fcgi?db=nucleotide&id=296010793) | 103 | 24.62 | 33.45 | >41 | 88 | 78 |
