# Supplementary figures and images for: Synoviocyte Derived-Extracellular Matrix Enhances Human Articular Chondrocyte Proliferation and Maintains Re-Differentiation Capacity at Both Low and Atmospheric Oxygen Tensions
Source: PLoS One. 2015 Jun 15;10(6):e0129961. doi: 10.1371/journal.pone.0129961 (PMC4468209; doi:10.1371/journal.pone.0129961)

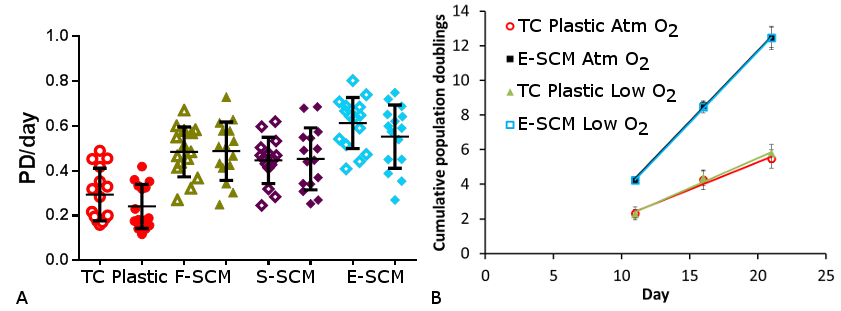

Supplement: S1 Fig — A) Comparison of population doubling rate between each surface at atmospheric (open symbols) and low (5%) oxygen tension (closed symbols). B) qPCR expansion data, recapitulating the increased expansion rate and lack of effect of oxygen on human chondrocyte expansion. (TIF) [file pone.0129961.s003.tif]

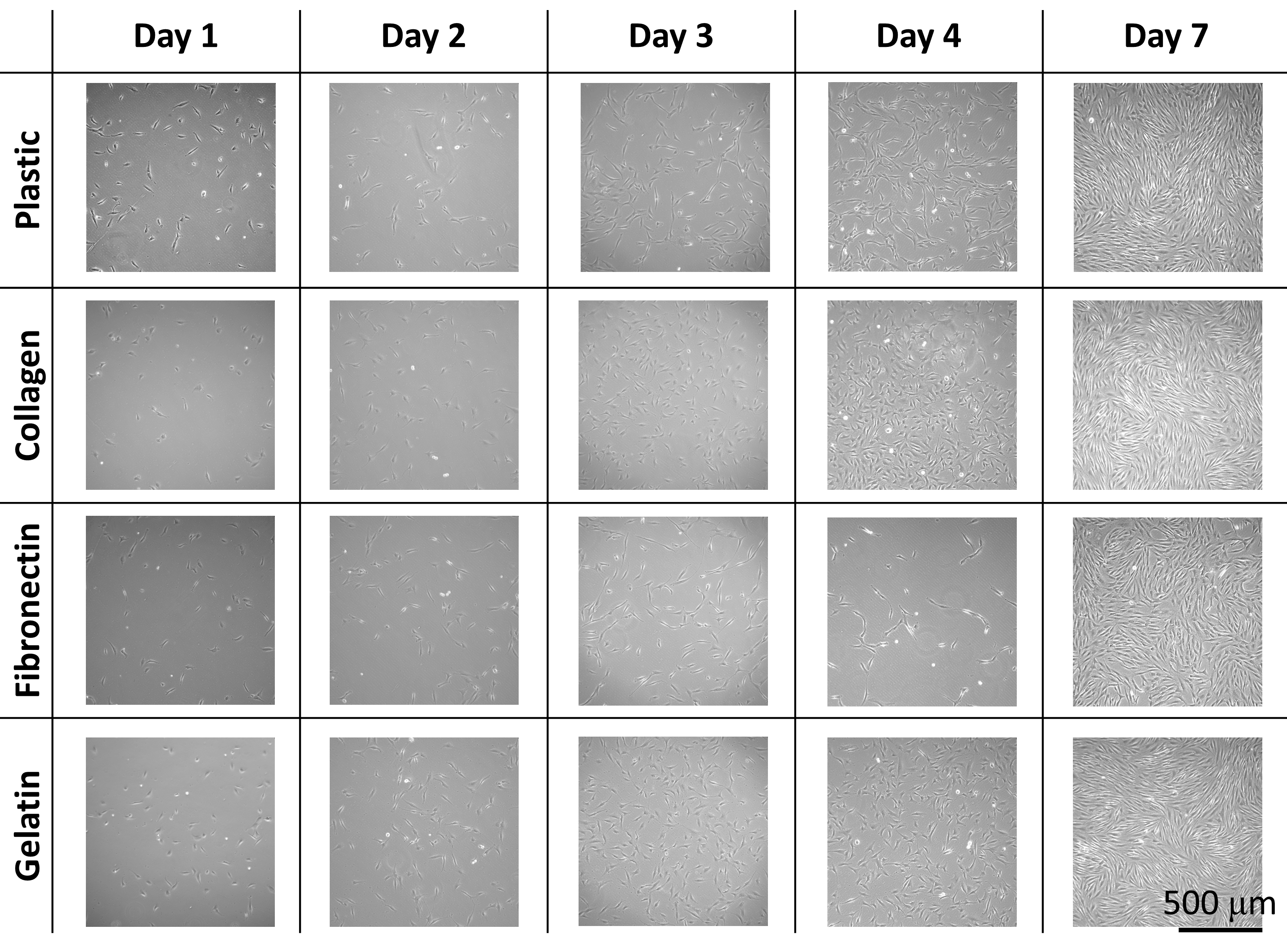

Supplement: S2 Fig — Analysis of growth and morphology on common surface coatings. Chondrocytes were seeded on each of the surfaces and tracked for 7 days. All images are at the same magnification (100x). (TIF) [file pone.0129961.s004.tif]

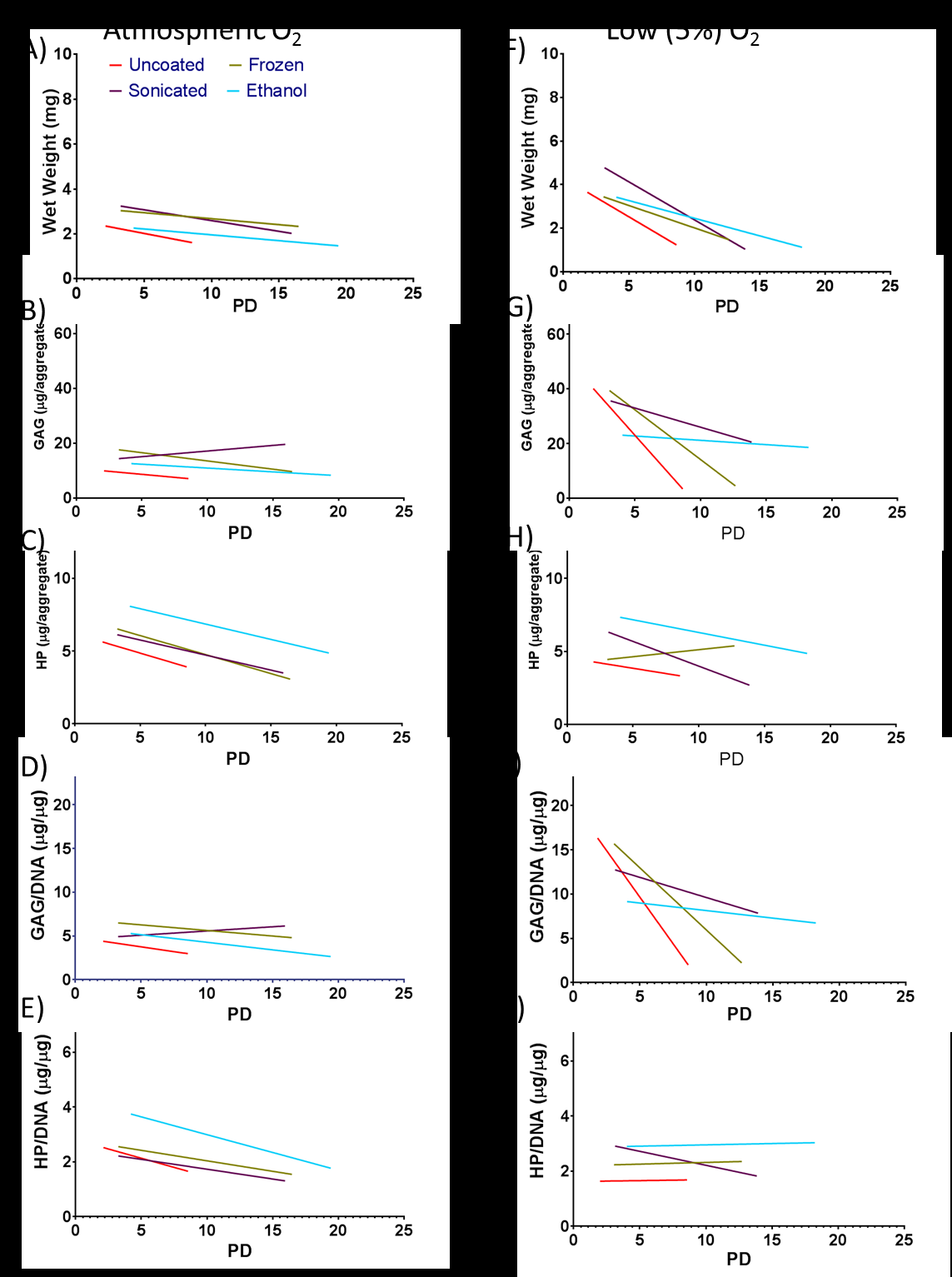

Supplement: S7 Fig — Regression analyses of biochemical measures against population doublings. Regressions were made combining all data from all 3 donors (n ≥ 23). A-E Atmospheric oxygen tension, F-J Low (5%) oxygen tension; A, F) Wet weight vs. population doublings; B,G) Total GAG (per aggregate) vs. population doublings; C,H) Total HP (per aggregate) vs. population doublings; D, I) Normalized GAG (GAG/DNA) vs. population doublings; E,J) Normalized HP (HP/DNA) vs. population doublings. (TIF) [file pone.0129961.s009.tif]

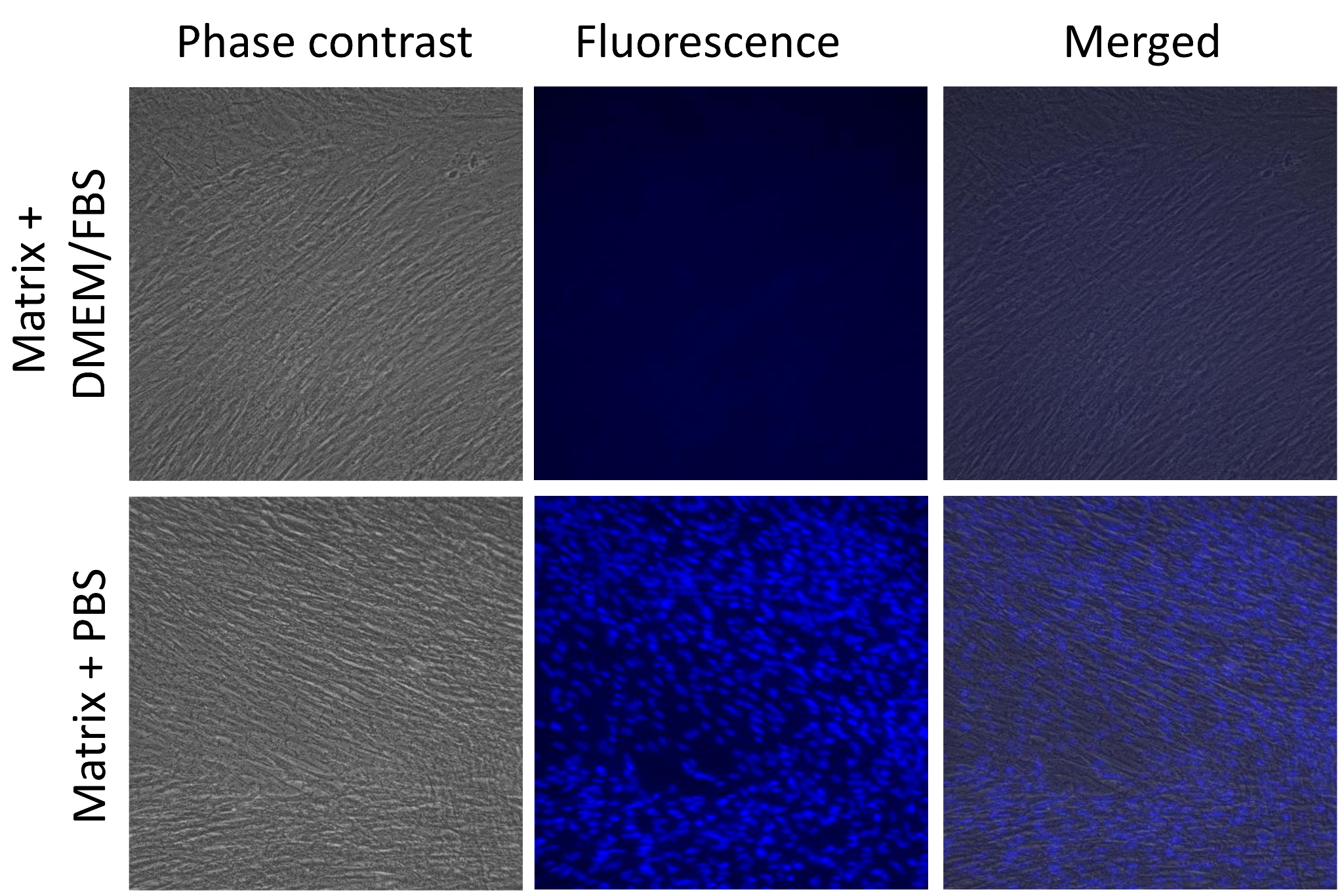

Supplement: S9 Fig — Synoviocyte-derived matrix was incubated with DMEM/FBS or PBS for 6 days; wells were then stained with Hoechst and imaged for nuclear staining (Fluorescence). (TIF) [file pone.0129961.s011.tif]
