# Supplementary material for: Synoviocyte Derived-Extracellular Matrix Enhances Human Articular Chondrocyte Proliferation and Maintains Re-Differentiation Capacity at Both Low and Atmospheric Oxygen Tensions
Source: PLoS One. 2015 Jun 15;10(6):e0129961. doi: 10.1371/journal.pone.0129961 (PMC4468209; doi:10.1371/journal.pone.0129961)
Supplement: S8 Fig — Representative sections of control tissues: rabbit (Rb) articular and auricular cartilage and human (Hu) articular cartilage. Representative sections from aggregates produced from cells expanded at atmospheric O2 on tissue culture plastic (uncoated) or E-SCM are also shown. (PPTX) [file pone.0129961.s010.pptx]

## Slide 1
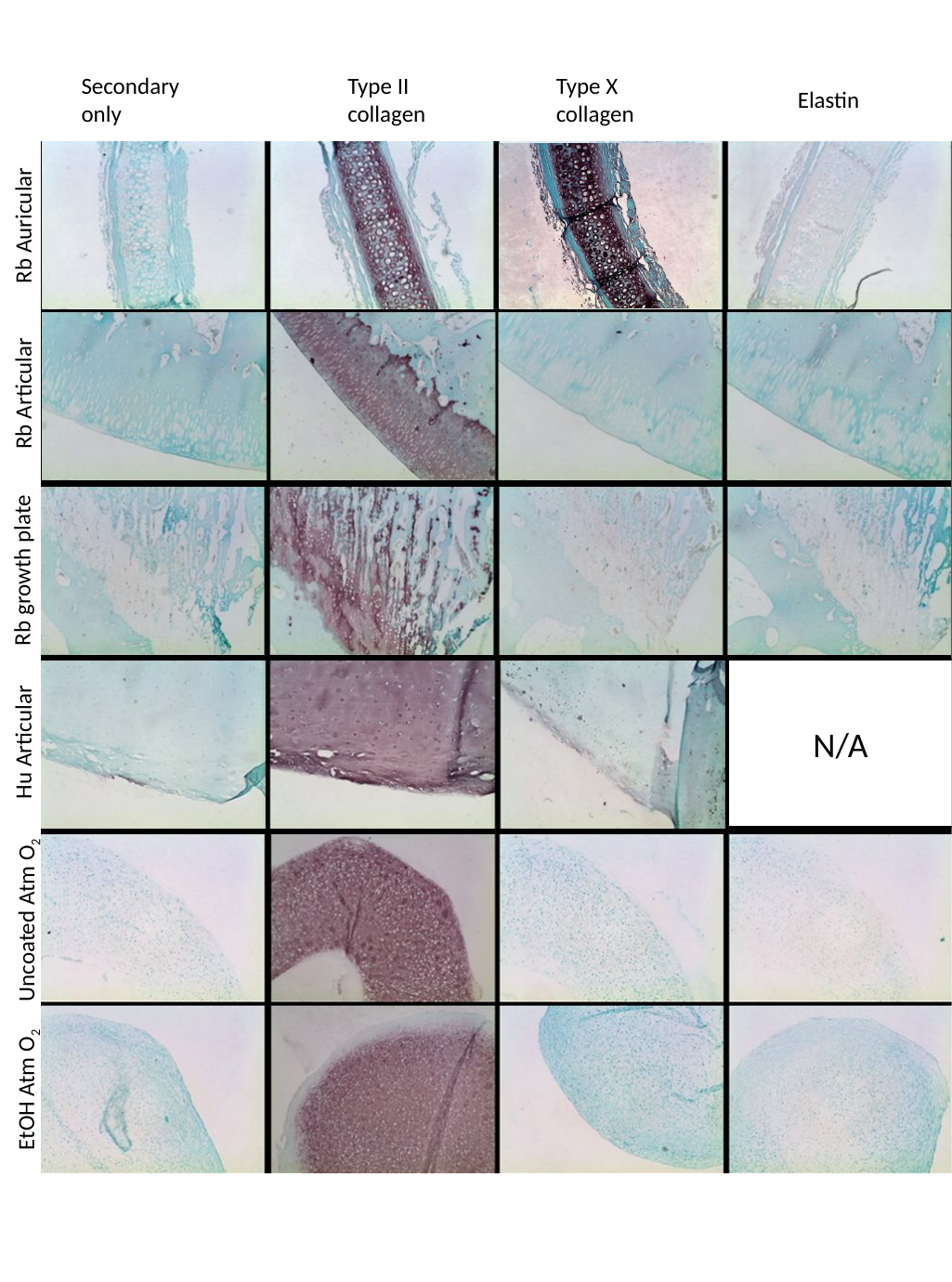

Secondary only
Type II collagen
Type X collagen
Elastin
Rb Auricular
Rb Articular
Rb growth plate
N/A
Hu Articular
Uncoated Atm O2
EtOH Atm O2
