# Supplementary figures and images for: Correction: Synoviocyte Derived-Extracellular Matrix Enhances Human Articular Chondrocyte Proliferation and Maintains Re-Differentiation Capacity at Both Low and Atmospheric Oxygen Tensions
Source: PLoS One. 2015 Sep 14;10(9):e0138409. doi: 10.1371/journal.pone.0138409 (PMC4569188; doi:10.1371/journal.pone.0138409)

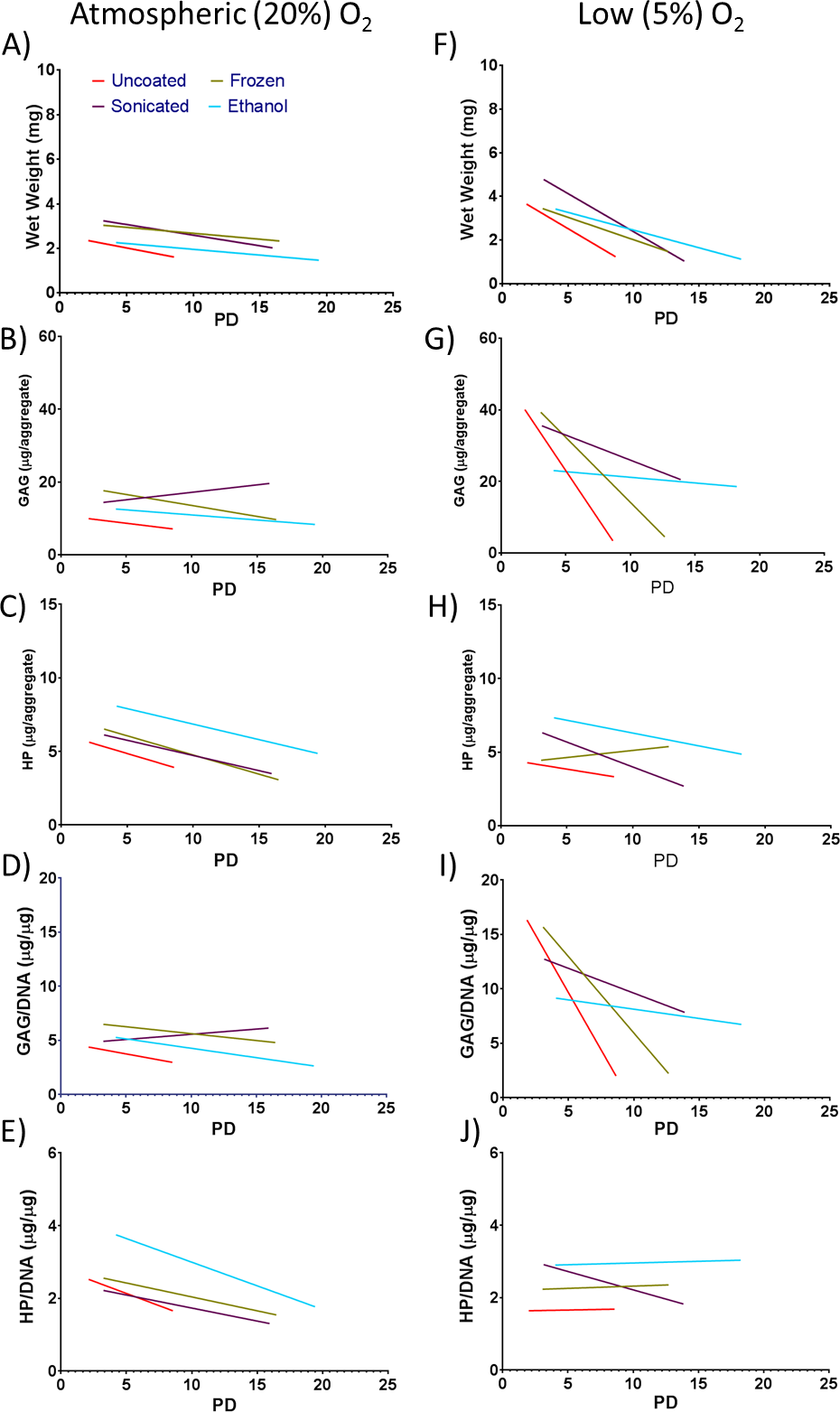

Supplement: S7 Fig — Regression analyses of biochemical measures against population doublings. Regressions were made combining all data from all 3 donors (n ≥ 23). A-E Atmospheric oxygen tension, F-J Low (5%) oxygen tension; A, F) Wet weight vs. population doublings; B,G) Total GAG (per aggregate) vs. population doublings; C,H) Total HP (per aggregate) vs. population doublings; D, I) Normalized GAG (GAG/DNA) vs. population doublings; E,J) Normalized HP (HP/DNA) vs. population doublings. (TIF) [file pone.0138409.s001.tif]
